# Suppressive Role of Androgen/Androgen Receptor Signaling via Chemokines on Prostate Cancer Cells

**DOI:** 10.3390/jcm8030354

**Published:** 2019-03-13

**Authors:** Kouji Izumi, Atsushi Mizokami

**Affiliations:** Department of Integrative Cancer Therapy and Urology, Kanazawa University Graduate School of Medical Science, 13-1 Takara-machi, Kanazawa, Ishikawa 920-8641, Japan; mizokami@staff.kanazawa-u.ac.jp

**Keywords:** prostate cancer, androgen receptor, castration-resistant prostate cancer, CCL2, CCL22, CCL5, migration

## Abstract

Androgen/androgen receptor (AR) signaling is a significant driver of prostate cancer progression, therefore androgen-deprivation therapy (ADT) is often used as a standard form of treatment for advanced and metastatic prostate cancer patients. However, after several years of ADT, prostate cancer progresses to castration-resistant prostate cancer (CRPC). Androgen/AR signaling is still considered an important factor for prostate cancer cell survival following CRPC progression, while recent studies have reported dichotomic roles for androgen/AR signaling. Androgen/AR signaling increases prostate cancer cell proliferation, while simultaneously inhibiting migration. As a result, ADT can induce prostate cancer metastasis. Several C-C motif ligand (CCL)-receptor (CCR) axes are involved in cancer cell migration related to blockade of androgen/AR signaling. The CCL2-CCR2 axis is negatively regulated by androgen/AR signaling, with the CCL22-CCR4 axis acting as a further downstream mediator, both of which promote prostate cancer cell migration. Furthermore, the CCL5-CCR5 axis inhibits androgen/AR signaling as an upstream mediator. CCL4 is involved in prostate carcinogenesis through macrophage AR signaling, while the CCL21-CCR7 axis in prostate cancer cells is activated by tumor necrotic factor, which is secreted when androgen/AR signaling is inhibited. Finally, the CCL2-CCR2 axis has recently been demonstrated to be a key contributor to cabazitaxel resistance in CRPC.

## 1. Introduction

Prostate cancer is among the most frequently diagnosed malignancies worldwide in men [1]. The five-year survival rate for localized prostate cancer is close to 100%, and the prognosis for localized prostate cancer is the best among all types of cancers; however, metastatic prostate cancer is associated with a very poor prognosis, with no curative treatments currently available [1,2]. Androgen/androgen receptor (AR) signaling is known to be a significant driver of prostate cancer progression, therefore androgen-deprivation therapy (ADT)—with or without anti-androgens—is often used as a standard form of care for patients with advanced and metastatic prostate cancer [3,4]. ADT has been demonstrated to improve not only serum prostate-specific antigen levels, but also patient survival, however prostate cancer generally progresses to castration-resistant prostate cancer (CRPC) following several years of ADT [5]. Several potential mechanisms underpinning CRPC progression that relate to AR function have been identified, including androgen hypersensitivity, AR mutation, ligand promiscuity, and AR variants. Nonetheless, no radical treatments exist at present and all AR-targeting agents for CRPC eventually fail to suppress cancer cell activity [6]. Recently, some studies have reported suppressive effects of androgen/AR signaling in prostate cancer cells, therefore suppression of AR function itself may cause CRPC [7,8]. Previously, we demonstrated that androgen/AR signaling increases prostate cancer cell proliferation, while simultaneously inhibiting cancer cell migration, which is induced by the activation of several C-C motif ligand (CCL)-receptor (CCR) axes downstream or upstream of androgen/AR signaling [9,10,11,12]. This review focuses on such suppressive effects of androgen/AR signaling on prostate cancer cells through CCL-CCR axes.

## 2. The Role of CCL2 as a Downstream Mediator of Androgen/AR Signaling

Therapeutic approaches that solely target androgen/AR signaling are insufficient to control prostate cancer cell activity [13,14,15]. Genetic ablation of AR in prostate epithelial cells promotes the development of invasive prostate cancer [7], suggesting that therapeutic suppression of androgen/AR function induces unwanted signals that may promote the progression of surviving prostate cancer cells to an advanced metastatic stage. When AR function of C4-2 (a human prostate cancer cell line) cells were silenced with AR-siRNA (siAR), using scramble RNA (scr) as a control, siAR cells were observed to possess an increased migratory capacity [8]. Cytokine array analysis of conditioned media from siAR and scr cells revealed increased CCL2 expression in siAR cells, supporting a potential role for prostate cancer cell-derived CCL2 in mediating local inflammatory responses during suppression of AR [8]. CCL2 is reported to play a potential role in stimulating capillary network formation of human microvascular endothelial cells in the microenvironment of prostate cancer [16]. C4-2 siAR cells were also observed to express increased levels of epithelial-mesenchymal transition (EMT) markers and pSTAT3 via the CCL2-CCR2 axis in an autocrine manner. In addition, C4-2 siAR cells were observed to possess significantly reduced levels of PIAS3 (the endogenous protein inhibitor of activated STAT3), which is controlled by androgen/AR signaling [17]. Notably, STAT3 activation was also observed to increase CCL2 expression levels in C4-2 siAR cells. These results suggest that androgen/AR signaling in prostate cancer cells may inhibit CCL2 and pSTAT3 expression through upregulation of PIAS3 [8,9]. EMT is believed to be an essential cancer cell characteristic for invasion and metastasis to distant sites [18]; pSTAT3 activation has been reported to play an important role in EMT induction, as well as inflammation and cancer progression [19,20]. Furthermore, ADT is known to be linked to EMT induction [21]. In summary, prostatic epithelial AR silencing via siAR promotes STAT3 activation and EMT in prostate cancer cells via CCL2 induction, which may be associated with a secretory phenotype and pro-invasive characteristics of prostate cancer cells [8,9].

## 3. The Role of CCL22 as a Further Downstream Mediator of CCL2

CCL2 is a powerful chemotactic protein for macrophages and tumor-associated macrophages (TAMs), which infiltrate into tumors and contribute to cancer progression via immune suppression [22,23]. CCL17 and CCL22, which are high-affinity ligands for CCR4, have both been reported to be secreted by TAMs, with immunosuppressive functions [24]. Correlations have previously been reported between CCR4 expression levels and metastasis in cancer cells [25,26]. Therefore, we aimed to elucidate the relationship between the CCL2-CCR2 axis and CCL17/22-CCR4 axis in prostate cancer progression. Both CCR2 and CCR4 were observed to be expressed in human prostate cancer cell lines and prostate cancer tissues; furthermore, in vitro co-culture of prostate cancer cells and macrophages resulted in increased CCL2 and CCR2 levels in prostate cancer cells [11]. Notably, addition of CCL2 induced both CCL22 and CCR4 expression in prostate cancer cells; CCL22 subsequently promoted the migration and invasion of prostate cancer cells in an autocrine manner, via enhanced phosphorylation of Akt [11]. The CCL22-CCR4 axis is known to chemo-attract regulatory T cells (Tregs) into tumor tissues; Tregs recognize self-antigens, including tumor antigens present in tumor tissues, and efficiently suppress the activation of tumor antigen-specific effector T cells [27]. In summary, CCL2 and CCL22 secretion in the prostate cancer tumor microenvironment may induce not only direct metastasis of prostate cancer cells, but also promote the activation of TAMs and Tregs, which facilitate a suitable environment for cancer progression.

## 4. The Role of CCL5 as an Upstream Mediator of Androgen/AR Signaling

Skeletal metastases occur in approximately 80% of patients with advanced prostate cancer, for which no curative treatment is available [28]. We previously reported that bone stromal cells and SaOS-2 osteoblast-like cells promote prostate cancer metastasis via activation of transforming growth factor-β1 (TGF β1) [29], which in turn induces the development of an immune suppressive microenvironment [30]. CCL2 is reported to increase bone metastasis through recruitment of TAMs and osteoclasts to the tumor site and blood vessel formation through vascular endothelial growth factor-A [31,32]. Therefore, we investigated whether further chemokines could be involved in the activation of prostate cancer cells within prostate cancer bone metastases. Migration of LNCaP cells (an AR-positive prostate cancer cell line) increased significantly when co-cultured with bone stromal cells isolated from prostate cancer bone metastases. Cytokine array analysis of conditioned media from bone stromal cell cultures subsequently identified CCL5, a high-affinity ligand of CCR5, as a concentration-dependent promoter of LNCaP cell migration [12]. LNCaP cell migration was observed to be suppressed by the addition of a CCL5-neutralizing antibody to cocultures with bone stromal cells, while AR knockdown using siRNA was observed to increase LNCaP cell migration compared with control cells [12]. As CCL5 was unable to promote migration of LNCaP siAR cells, it was concluded that elevated CCL5 secretion by bone stromal cells from metastatic lesions induced prostate cancer cell migration in a CCL5-dependent manner, upstream of AR signaling [12]. Upregulation of CCL5 has previously been reported to increase the aggressive potential of breast cancer cells and the invasiveness of prostate cancer cells [33,34,35]. In addition, Luo et al. found that CCL5 upregulation in bone marrow mesenchymal stem cells increased the metastatic potential of prostate cancer cells, and subsequently downregulated AR signaling, due to inhibition of AR nuclear translocation [36]. Furthermore, CCL5 has been found to suppress prolyl hydroxylase expression, leading to suppression of VHL-mediated HIF2α ubiquitination and suppression of AR signaling [37]. Results obtained using LNCaP siAR cells indicate that CCL5 activity is located upstream of AR signaling. Moreover, SaOS-2 did not promote the migration of PC-3 AR-negative prostate cancer cells [12]. These results suggest that the migratory potential of AR-positive prostate cancer cells in bone metastases is increased by CCL5, secreted by bone stromal cells via the suppression of androgen/AR signaling. CCL5 is also secreted by prostate cancer-associated fibroblasts and recruited macrophages into the prostate cancer microenvironment [38]. Estrogen receptor α could reduce prostate cancer cell invasion through reduction of CCL5 secretion from fibroblasts and macrophage infiltration prostate cancer [38].

## 5. Treatment Strategies Targeting CCL-CCR Axes and Androgen/AR Signaling

### 5.1. CCL2-CCR2 Axis

A new role for AR silencing in the mediation of EMT induction via activation of the CCL2-CCR2 axis in the tumor microenvironment provides new therapeutic targets for preventing potential prostate cancer metastasis at later stages. A previous study reported on treatment of forty-six CRPC patients with the human CCL2 monoclonal antibody, carlumab, in a phase 2 trial. Unfortunately, this single-arm study was not able to meet its primary objective to demonstrate potential therapeutic benefits of carlumab alone in patients with metastatic CRPC who had failed prior docetaxel-based treatment [39]. Carlumab was unable to sustain durable free CCL2 suppression, permitting rapid rebound and increases in CCL2 to baseline or higher concentrations; this insufficient suppression meant meaningful clinical responses could not be achieved [39]. To entirely suppress the CCL2-CCR2 axis, a receptor antagonist may provide a more suitable treatment method as receptor blockade efficiency is irrespective of serum CCL2 concentrations. Several CCR2 antagonists has been reported in the literature [40,41]. As stated in Section 2, CCL2-CCR2 axis and STAT3 activate each other in prostate cancer cells, therefore STAT3 is also regarded as a potential treatment target. We confirmed inhibition of STAT3 activity by a STAT3 inhibitor, AG490, resulted in down regulation of EMT gene expression in C4-2 siAR cells [8]. 

### 5.2. CCL22-CCR4 Axis 

Tissue microarray analysis revealed a correlation between staining intensity of CCR4 and prostate cancer progression, however no such correlation existed with CCR2, despite the fact that CCR2 and CCR4 intensities were correlated with one another. Therefore, the CCL22-CCR4 axis may prove a more significant driver of prostate cancer migration and invasion than the CCL2-CCR2 axis [11]. Phosphorylation of Akt proteins is more effectively inhibited by CCR4 antagonists than CCR2 antagonists, further indicating the efficiency of CCR4 antagonist therapy against prostate cancer migration and invasion [11]. Akt activation is controlled by phosphorylation of the two key residues threonine 308 (Thr308) and serine 473 (Ser473) [42], and their phosphorylation promotes prostate cancer cell growth, proliferation, motility, and survival [43,44,45]. Our previous results indicated that the CCL22-CCR4 axis controls phosphorylation of Ser473. It has been previously been demonstrated that CCL2 promotes prostate cancer cell proliferation, migration, and survival via Akt-activation-dependent mechanisms [46,47,48]. Taken together, these results indicate that the CCL22-CCR4 axis may prove to be a better therapeutic target than the CCL2–CCR2 axis for prostate cancer patients. CCR4 expression is observed on tumor cells derived from the majority of adult T-cell leukemia-lymphoma (ATL) patients, therefore mogamulizumab, an anti-CCR4 antibody, has been approved in Japan for the treatment of relapsed/refractory ATL [49,50]. In addition, potent inhibition of Akt signaling using an Akt inhibitor was associated with a tolerable safety profile and meaningful disease control in a subgroup of patients with solid tumors during phase 1 and 2 trials [51,52]. Application of such CCL22-CCR4 targeting agents for therapy of CRPC patients is expected in the near future.

### 5.3. CCL5-CCR5 Axis and Others

In a recent study of ours that focused on the effects of coffee compounds on prostate cancer cells, the coffee diterpenes kahweol acetate and cafestol were observed to synergistically inhibit prostate cancer cell proliferation and migration [53]. These diterpenes were capable of inhibiting androgen/AR signaling without inducing prostate cancer cell secretion on CCL2 and CCL5 [53]. It is noteworthy that expression of CCR2 and CCR5, receptors for CCL2 and CCL5, respectively, visibly decreased following diterpene administration in prostate cancer cells [53]. Kahweol acetate and cafestol may, therefore, represent potential therapeutic candidates, especially in combination therapy for the treatment of both castration-sensitive prostate cancer and CRPC [53].

## 6. CCL Involvement Various Pathways of Prostate Cancer Progression

### 6.1. Carcinogenesis

Co-culturing of immortalized prostate epithelial cells with macrophages has been observed to induce prostate tumorigenesis, involving the signaling alteration of macrophage AR-inflammatory chemokine CCL4-pSTAT3 activation, EMT, and p53/PTEN tumor suppressor down-regulation [54]. Furthermore, in vivo studies have demonstrated that PTEN(+/−) mice lacking macrophage AR develop fewer prostatic intraepithelial neoplasia lesions, supporting an important role for macrophage AR signaling during prostate tumorigenesis [54]. CCL4-neutralizing antibodies effectively inhibited macrophage-induced prostate tumorigenic signaling, while CCL4 upregulation was associated with increased Snail expression and p53/PTEN down-regulation in high-grade prostatic intraepithelial neoplasia and prostate cancer [54]. This study identified the AR-CCL4-pSTAT3 axis is a key regulator during prostate tumorigenesis and highlighted the important roles of infiltrating macrophages and inflammatory cytokines during prostate tumorigenesis [54,55].

### 6.2. Lymph Node Metastasis

Tumor necrotic factor (TNF) is negatively regulated by androgen/AR signaling, and androgen/AR signaling blockade induces TNF mRNA in prostatic stroma [56]. We observed that human prostate cancer cells (PC-3, DU145, LNCaP, and LNCaP-SF) express both TNF-α and CCR7 and that low concentrations of TNF-α can induce CCR7 expression in prostate cancer cells through phosphorylation of extracellular signal-regulated kinases in an autocrine manner [57]. CCL21, a ligand of CCR7 that is secreted by fibroblastic reticular cells in lymph nodes and is abundant in the T-cell zone of the lymph node [58], was found to promote prostate cancer cell migration via protein kinase p38 phosphorylation [57]. These results suggest that TNF-α induces CCR7 expression and that the CCL21-CCR7 axis is capable of increasing the metastatic potential of prostate cancer cells during lymph node metastasis. In combination with ADT, the CCL21-CCR7 axis may, therefore, prove a superior target compared with single androgen/AR signaling-targeted therapy for treatment of patients with prostate cancer and lymph node metastasis.

### 6.3. Resistance to Taxanes

Understanding the underlying mechanisms behind chemoresistance and disease progression in patients with prostate cancer is important in order to develop novel treatment strategies. In particular, cabazitaxel resistance is a considerable challenge in CRPC patients as cabazitaxel is often administered as a last resort [59]. The mechanism through which cabazitaxel resistance develops is still unclear, however. We previously established a cabazitaxel-resistant prostate cancer cell line, DU145-TxR/CxR, from a paclitaxel-resistant cell line, DU145-TxR [60]. The cDNA microarray analysis revealed that CCL2 expression was upregulated in both DU145-TxR and DU145-TxR/CxR cells, compared with control DU145 cells. Furthermore, the secreted CCL2 protein level in DU145-TxR and DU145-TxR/CxR cells was observed to be higher than in the parental DU145 cells [61]. Stimulation of DU145 cells with CCL2 increased proliferation during cabazitaxel treatment, while CCR2 antagonist suppressed the proliferation of DU145-TxR and DU145-TxR/CxR cells during cabazitaxel treatment [61]. The CCL2-CCR2 axis was found to reduce apoptosis through inhibition of caspase-3 and poly(ADP-ribose) polymerase (PARP), indicating that CCL2 is potentially a key contributor to cabazitaxel resistance in prostate cancer cells [61]. Inhibition of the CCL2-CCR2 axis may provide a potential therapeutic strategy against both chemosensitive CRPC and chemoresistant CRPC.

## 7. Concluding Remarks

**Figure 1:**
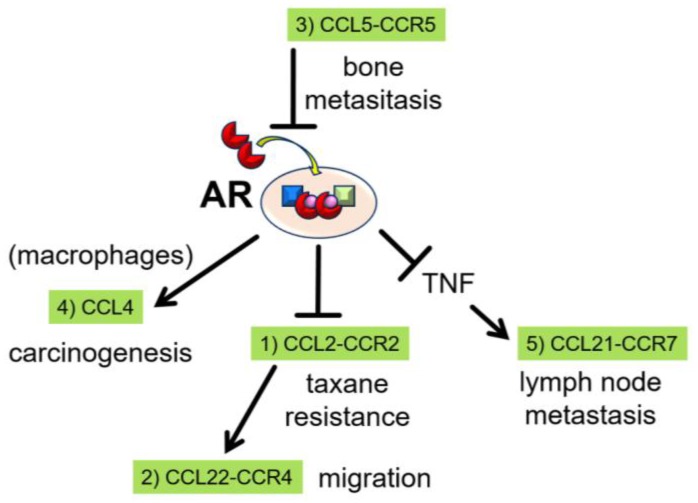
Androgen receptor (AR) and C-C motif ligand (CCL)-receptor (CCR) axes. (1) Androgen/AR signaling negatively regulates CCL2 secretion and inhibits prostate cancer cell migration. The CCL2-CCR2 axis contributes to chemoresistance to taxanes. (2) The CCL22-CCR4 axis is located downstream of the CCL2-CCR2 axis and increases the migratory capacity of prostate cancer cells. (3) CCL5 activity occurs upstream of AR signaling and increases the migratory capacity of prostate cancer cells via inhibition of androgen/AR signaling. (4) CCL4 is positively regulated by androgen/AR signaling in macrophages, and is a key regulator during prostate tumorigenesis. (5) TNF, which is negatively regulated by androgen/AR signaling, induces CCR7 expression in prostate cancer cells. CCR7 subsequently binds CCL21 from fibroblastic reticular cells in lymph node, resulting in increased migratory capacity.

Our recent studies have elucidated several CCL-CCR axes involved in prostate cancer progression, some of which are negatively regulated by androgen/AR signaling and vice versa (Figure 1). Other CCL-CCR axes may play significant roles in prostate cancer progression, while CCL-CCR axes form chemokine-networks in which CCL-CCR axes are capable of activating and/or inactivating one another. In summary, the complete elucidation of this chemokine-network, including the exact function of each chemokine, is required to control prostate cancer cells across every stage.

## Abbreviations

AR	androgen receptor
ADT	androgen-deprivation therapy
CRPC	castration-resistant prostate cancer
CCL	C-C motif ligand
CCR	CCL-receptor
EMT	epithelial-mesenchymal transition
siAR	AR-siRNA
scr	scramble RNA
TAMs	tumor-associated macrophages
Tregs	regulatory T cells
TGF β1	transforming growth factor-β1
Thr308	threonine 308
Ser473	serine 473
ATL	adult T-cell leukemia-lymphoma
TNF	tumor necrotic factor
PARP	poly(ADP-ribose) polymerase
